# The global burden of erectile dysfunction and its associated risk factors in diabetic patients: an umbrella reviews

**DOI:** 10.1186/s12889-024-20300-7

**Published:** 2024-10-14

**Authors:** Tegene Atamenta kitaw, Biruk Beletew Abate, Befkad Derese Tilahun, Gizachew Yilak, Moges Beriye Rede, Addisu Getie, Ribka Nigatu Haile

**Affiliations:** 1 Department of Nursing, College of Health Science, Woldia University, Woldia, Ethiopia; 2 School of Medicine, College of Health Science, Woldia University, Woldia, Ethiopia; 3 Department of Nursing, College of Medicine and Health Sciences, Debre Markos University, Debre Markos, Ethiopia

**Keywords:** Global burden, Erectile dysfunction, Associated risk factors, Diabetic patients

## Abstract

**Background:**

Erectile dysfunction is no longer a whisper in the shadows; it’s a rising tide threatening the sexual health of millions of men in different regions. In the cases of diabetes, the condition worsens and has a potent cocktail of physical and psychological distress, chipping away at men’s confidence, self-esteem, and mental health. This worrying trend shows no signs of slowing down, with projections claiming a staggering 322 million men globally could be affected in the near future. This urgent issue demands immediate attention and action. Thus, this umbrella review intended to estimate the current burden of erectile dysfunction and associated risk factors among diabetic patients in the global context.

**Methods:**

Following PRISMA guidelines, we searched for relevant studies in PubMed, Embase, Scopus, Web of Science, Cochrane Database of Systematic Reviews, and Google Scholar. These studies reported the prevalence of erectile dysfunction and associated risk factors in diabetic patients. The quality of the included studies was assessed using the Assessment of Multiple Systematic Reviews 2 tool. To estimate the pooled prevalence of erectile dysfunction, we employed a weighted inverse variance random-effects model. We further conducted subgroup analyses, assessed heterogeneity and publication bias, and performed sensitivity analyses to strengthen the robustness of our findings. Prediction intervals were also calculated to estimate the range within which future observations will likely fall. In all statistical analyses, the statistical significance was declared at P- value < 0.05.

**Results:**

In this umbrella review, a total of 108 030 male diabetic patients were included to estimate the global prevalence of erectile dysfunction. The pooled global prevalence of erectile dysfunction in diabetic patients was 65.8% (95CI: 58.3 − 73.3%), while In Africa it was 62.9% (95CI: 46.1–79.7). Age (> 40 years) (AOR = 1.95, 95CI: 1.03–3.24), DM duration (> 10years) (AOR = 1.90,95CI: 1.16–2.65), peripheral vascular disease (AOR = 2.74, 95CI: 1.42–4.06) and BMI (> 30 kg/m2) (AOR = 1.07,95CI: 1.01–1.20) were identified as associated risk factors of erectile dysfunction in diabetic patient.

**Conclusion:**

The high global prevalence of erectile dysfunction (ED) in diabetic patients is alarming, with an estimated two-thirds experiencing the condition. These findings underscore the significant burden of ED faced by diabetic men and emphasize the urgent need for global attention to the issue. This includes promoting early screening for erectile dysfunction in this population and ensuring access to appropriate treatment and support.

**Supplementary Information:**

The online version contains supplementary material available at 10.1186/s12889-024-20300-7.

## Introduction

The number of men who have erectile dysfunction (ED) is on the rise worldwide, posing a growing threat to their overall well-being. This concerning trend is expected to continue, with projections indicating a staggering 322 million men globally [1], highlighting the urgency of addressing this issue. ED is referred to as a consistent inability to obtain or maintain an erection that is sufficient for sexual satisfaction. It also includes having satisfactory or complete penetrative intercourse [2].

Several chronic conditions, including diabetes mellitus (DM), cardiovascular disease, and depression, exhibit a strong correlation with increased rates of erectile dysfunction (ED), with DM playing a particularly significant role [3]. In diabetes, erectile dysfunction stems from multiple mechanisms. Chronically high blood sugar levels lead to damage in blood vessels (endothelial dysfunction) [4], buildup of harmful sugar byproducts (advanced glycation end products), increased cell waste and damage (oxidative stress), and malfunctioning nerves (neuropathy). These factors disrupt the normal erectile response, making it difficult to achieve and maintain an erection [5]. Diabetes can cause two types of nerve damage, peripheral and autonomic, that both contribute to erectile dysfunction (ED). Peripheral neuropathy adversely affects signals between the penis and the brain, making it harder for the body to become stimulated [6]. It also weakens the muscles that control blood flow in the penis, making it difficult to get and keep an erection [7]. Cardiovascular disease (CVD) can also lead to erectile dysfunction (ED) by impairing penile blood flow. Conditions like hypertension, diabetes, and atherosclerosis associated with CVD restrict the ability of penile blood vessels to dilate properly, contributing to ED [8].

Erectile dysfunction is more than just a physical issue; it can significantly impact a man’s mental and emotional well-being, affecting his confidence, relationships, and overall happiness [9]. ED in people with diabetes may signal silent heart disease and predict future cardiovascular events while also impacting mental well-being by lowering self-esteem and increasing anxiety and depression [10]. Erectile dysfunction, while not directly life-threatening, can cause significant distress and impact quality of life. Along with the stigma surrounding the problem, it often leads to underreporting. Thus, early detection and management of factors contributing to ED remain challenging [11].

By 2025, an estimated 322 million men will have ED, underscoring its critical public health impact [12]. Among diabetic patients, ED prevalence ranges from 35 to 90% [13, 14]. ED leads to diminished quality of life, anxiety, strained relationships, and economic consequences like higher absenteeism and reduced productivity [15, 16]. Studies show men with ED miss work more frequently and experience greater work impairment compared to colleagues without ED [17]. However, taking control of the underlying condition can help alleviate some of these challenges.

Erectile dysfunction (ED) is a common complication of diabetes, affecting millions of men worldwide. Yet, despite its significant impact on diabetic patients, it remains largely a hidden burden, shrouded in stigma and shame. This lack of awareness and open discussion has far-reaching consequences, hindering early detection, treatment, and, ultimately, the prevention of long-term complications. Thus, understanding erectile dysfunction among diabetic patients in a global context can have contribute to improving the health and well-being of diabetic patients worldwide. It is also essential to gain global attention, promoting global collaboration to prevent the silent burden. Thus, this umbrella review aims to estimate the burden of erectile dysfunction and associated risk factors in diabetic patients in a global context.

## Methods

### Protocol development and registration

This umbrella protocol was designed in accordance with preferred methods of reviewing available Systematic Review and Meta-analysis (SRM) studies [18]. First, the existence of similar umbrella review was cheeked on PROSPERO, found that there are no similar studies registered. Then, the protocol of this umbrella review was summited and registered (CRD42023488922) in PROSPERO. PROSPERO registration related information can be available upon the reasonable request of the primary author. This umbrella review focus on a systematic synthesis of existing systematic review and meta-analysis studies towards the prevalence of erectile dysfunction and associated risk factors in diabetic patients worldwide.

### Searching strategy and information sources

A compressive literature search was conducted regarding systematic review and meta-analysis of the prevalence of erectile dysfunction and associated risk factors in diabetic patients on Embase, Web of Sciences, PubMed, Cochrane Database of Systematic Reviews, Scopus, International Scientific Indexing (ISI), and Google Scholar using the PICO frameworks. Combinations, keywords and MeSH term were employed to retrieve the studies. Besides, snowballing technique was employed to retrieve additional studies in citation list of articles found in available database. Grey literature and manual search were also done find unindexed/not published/ research articles. Search strategies were drafted using concepts and key search terms. The first concept: Erectile dysfunction: “erectile dysfunction”, “erectile problem”, “impotency”, “impotence” “sexual impotence” and “sexual dysfunction”. The second concept: Risk factors: “risk factors”, “associated factors”, “determinants”, “predictors” and “cause”. The third concept: Diabetic: “diabetic”, “diabetes”, “diabetes mellitus” and “DM”. The fourth concept: (SRM): “meta-analysis’, ‘systematic review’, and ‘review’. Literature searching was conducted the two authors (TAK and RNH), independently. Any inconsistency was resolved by agreement. In the case of article with incomplete information, the primary authors of the respective article was contacted. *We used the search terms independently and/or in combination using “OR” or “AND”. An example of a research string for the PubMed database was as follows: ((((((((erectile dysfunction[Title/Abstract]) OR (erectile problem[Title/Abstract])) OR (impotency[Title/Abstract])) OR (impotence[Title/Abstract])) OR (sexual impotence[Title/Abstract])) OR (sexual dysfunction[Title/Abstract])) AND (((((risk factors[Title/Abstract]) OR (associated factors[Title/Abstract])) OR (determinants[Title/Abstract])) OR (predictors[Title/Abstract])) OR (cause[Title/Abstract]))) AND ((((diabetic[Title/Abstract]) OR (diabetes[Title/Abstract])) OR (diabetes mellitus[Title/Abstract])) OR (DM[Title/Abstract]))) AND (((meta-analysis[Title/Abstract]) OR (systematic review[Title/Abstract])) OR (review[Title/Abstract])).*

Besides, ‘related article’ and ‘citied by’ features of PubMed database were used to find article from the included studies.

### Eligibility criteria

#### Inclusion criteria

A systemic review and meta-analysis studies with reported prevalence and/or at least one associated risk factors of erectile dysfunction among diabetic patients written in English language was included. For respective SRM to be considered of this umbrella review, it should fulfill the following prioritized criteria. (1) presented a defined literature search strategy, (2) appraised included studies using a relevant tool, and (3) followed a standard approach in pooling studies and providing summary estimates.

#### Exclusion criteria

Articles were excluded in one of the following reasons: (1) article did not measure the outcome of interest for this umbrella review, (2) article written other than in English language, and (3) narrative reviews, expert opinions, case reports, editorials, correspondence, abstracts, and methodological studies.

### Data extraction and management

Two reviewers (TAK and RNH) conducted data extraction independently using a standardized extraction form. Screening and selection of article were first done though title and abstract, then after reviewing of full text was done. In the cases of disagreement, discussion with other reviewers was made to decide for the final selection of article to include in this umbrella review. After systemic search was done, potentially eligible article was imported to EndNote 21. Duplicated studies were removed in conditions where two or more articles have shared common characteristics. Structured data extraction in the form of Microsoft Excel spreadsheet was prepared and used. The extracted data form includes: (1) study identification (last name of the primary author and year of publications), (2) the aim and the type of review, (3) prevalence of erectile dysfunction, (4) odds ratio and 95%CI of risk factors of erectile dysfunction, (5) the number of include primary studies within SRM, (6) the number of sample size included, (7) publication bias and quality assessment technique, (8) analytic model type (fixed effect/ random) and (9) the final conclusion of the SRM.

### Quality assessment

The quality of the included article was reviewed by two independent reviewers using the Assessment of Multiple Systematic Reviews (AMSTAR 2) tool. The new quality assessment tool extended from the previous AMSTAR. It contains 16 items to measure the whole approaches of the specific review of study. The AMSTAR 2 tool is stronger, aggressive and minimize quality scoring bias than the previous AMSTAR. The specific included article was extracted based on the 16 times of AMSTAR 2 tool. The 16 items of the article was filled to the online system in AMSTAR.com. The AMSTAR 2 automatically classify the level of evidence(quality) of specific SMR in to four categories: (a) High quality evidence, (b) moderate quality evidence, (c) low quality evidence and (d) critically low-quality evidence. Article with critically low-quality evidence was not included in this umbrella review [19].

### Statistical analysis

Once the data extraction was completed in Microsoft Excel format, the data was imported to STATA version 17 software for analysis. Qualitative and narrative methods was employed to the present the summarized estimate of included studies. In a condition of two or more estimate on the same topic was found, the range of estimate and or pooled estimate was used. Standard error was computed by considering a binomial distribution formula. The overall prevalence of erectile dysfunction was pooled using random effect model [20]. Besides, the pooled prevalence estimates and associated risk factors of erectile dysfunction were presented by using forest plot. Cochrane’s Q statistics (Chi-square), invers variance (I^2^) and p-values [21] were computed to show the level of heterogeneity between studies. Zero invers variance (I^2^) was revealed a true homogeneity, whereas 25%, 50% and 75% shows a low, moderate and high heterogeneity, respectively [22, 23]. Subgroup analysis was done using, publication year, sample size, study quality (AMSTAR 2), setting, and number of included study. Leave one out (sensitivity) meta-analysis was done to see the effect of single study on overall pooled estimation. Funnel plot and Egger’s regression test was computed to identify the publication bias [24].

## Results

**Fig. 1:**
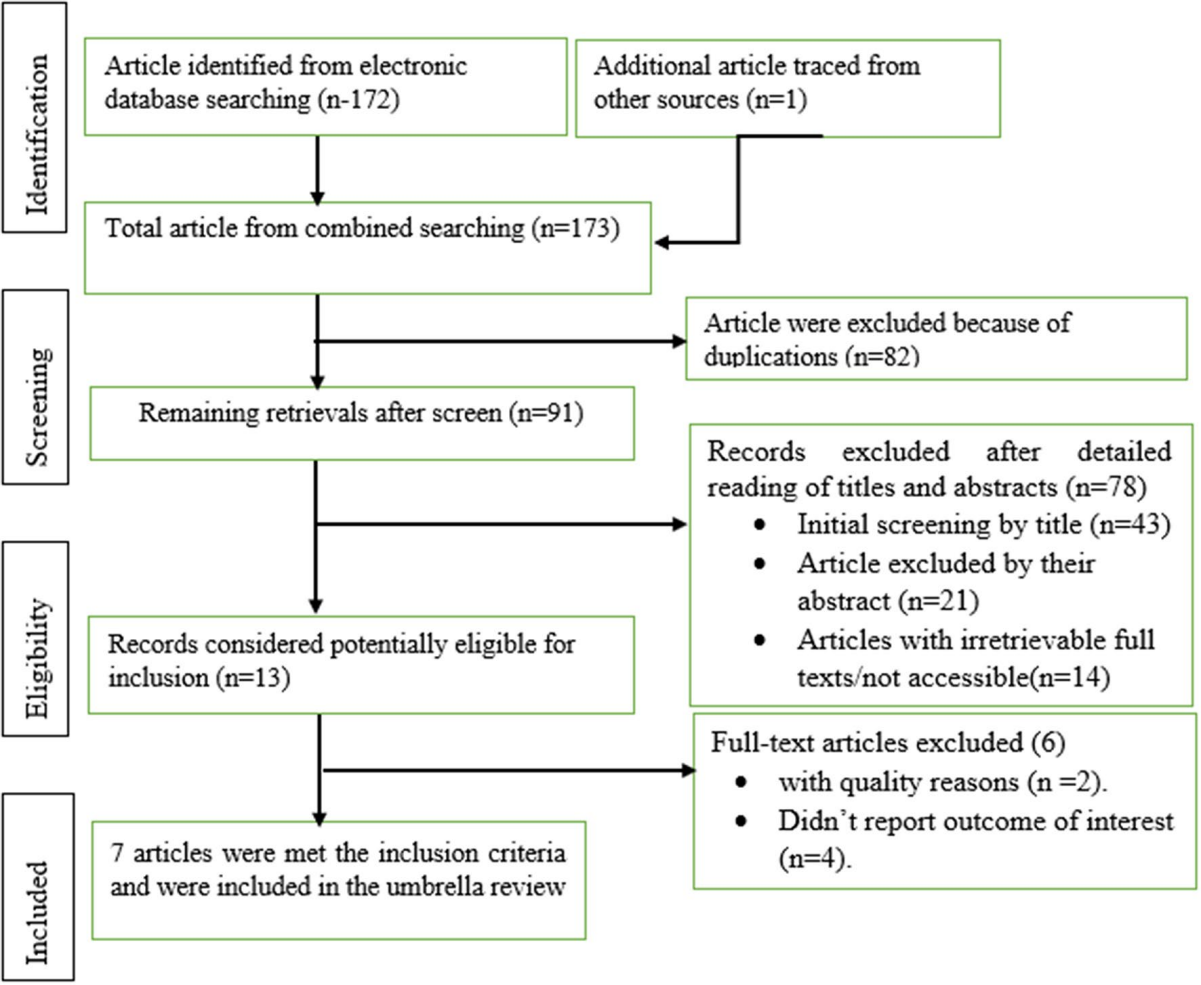
Flow chart diagram describing selection of studies for umbrella review the systematic review and meta-analysis of global burden of erectile dysfunction in diabetic, 2023

A total of 173 records were retrieved from different database search engines and traced other sources. 82 of them were excluded because of duplications through the EndNote citation manager. From 91 records, 78 retrievals were excluded after detailed reading of titles and abstracts. The remaining 13 records were potentially eligible for inclusion. After thoroughly checking the full publications of 13 articles, 5 studies were removed because their outcome estimates varied from the outcome of interest. Finally, 7 eligible studies were included in this umbrella review of systematic review and meta-analysis to estimate the global burden of erectile dysfunction in diabetic patients [12, 25–30]. Out of seven studies, five reported on the prevalence of erectile dysfunction in diabetic patients. (Fig. 1).

### Characteristics of original studies

All included reviews were published from 2017 onwards and included only primary studies that were published from 1980 to 2020. The number of primary studies included in each of the reviews ranged from 5 (with 2161 male diabetic participants) to 145 (with 88,577 male diabetic participants). In this umbrella review, a total of 108, 030 male diabetic patient were included to estimate the global prevalence of erectile dysfunction. Two of the studies were from all continent, one study was from Africa and the remains were from specific countries (China and Ethiopia). PubMed, MEDLINE and Embase were the most cited data bases for searching primary studies. Four of the studies use a standard quality assessment criterion to assess the quality of primary studies. (Table 1).

**Table 1:** Summarizes the key characteristics of the included review studies in this umbrella review,2023

Study characteristics	Authors						
	Kouidrat et al., [25]	Shiferaw et al., [28]	Tang et al., [26]	Wang et al., [27]	Weldesenbet et al., [29]	Shiferaw et al., [28]	Yamada et al., [30]
Publication Year	2017	2020	2023	2018	2021	2020	2012
Review years	1980 to 2016	2006–2014	2001–2020	2000–2018	2010–2017	2006–2019	1995–2011
Setting	Global	Africa	China	Global	Ethiopia	Africa	Global
Data base searched	PubMed, EMBASE and SCOPUS	PubMed, Web of Science, Cochrane Library, Scopus, African Journals Online, and Google Scholar	China national knowledge internet (CNKI), Wanfang database, Pubmed, and Embase	PubMed, MEDLINE, Embase, and Web of Science	PubMed, ScienceDirect, Google Scholar	PubMed, Web of Science, Scopus, African Journals Online, Wiley Online Library and Google Scholar	MEDLINE and the Cochrane Library
Number of studies	145	13	18	5	6	17	12
Sampl size	88,577	3501	11,424	2525	2003	6002	22,586
ED Prevalnce(%)	59.10%	71.44%	69.78%	74.22%	54.32%		
Quality assessment	Quality assessed but not used standard criteria	Newcastle–Ottawa quality	AHRQ	Newcastle-Ottawa scale	Newcastle–Ottawa Scale	Newcastle-Ottawa Scale	Newcastle–Ottawa Scale
AMSTAR score 2	Moderate	High	High	High	Moderate	High	Low

### Quality of the included studies

All studies were assessed using the AMSTAR 2 quality assessment tool for systemic review and meta-analysis. AMSTAR 2 is a newly developed quality assessment technique from AMSTAR 1. The newly emerged tool contains 16 items, which differ from the previous AMSTAR (includes 11 items). We assessed the quality of studies online at AMSTRA.com, which generated the overall evidence of the quality of the review. Based on the 16 items, AMSTAR 2 classifies the review article into four categories: High, moderate, low, and critical low appraisal. In this study, according to the AMSTAT 2 decision of quality of the review, 4 studies were high quality, 2 were moderate, and 1 was low quality. (Supplementary file 1).

### Prevalence of erectile dysfunction in diabetic patients

**Fig. 2:**
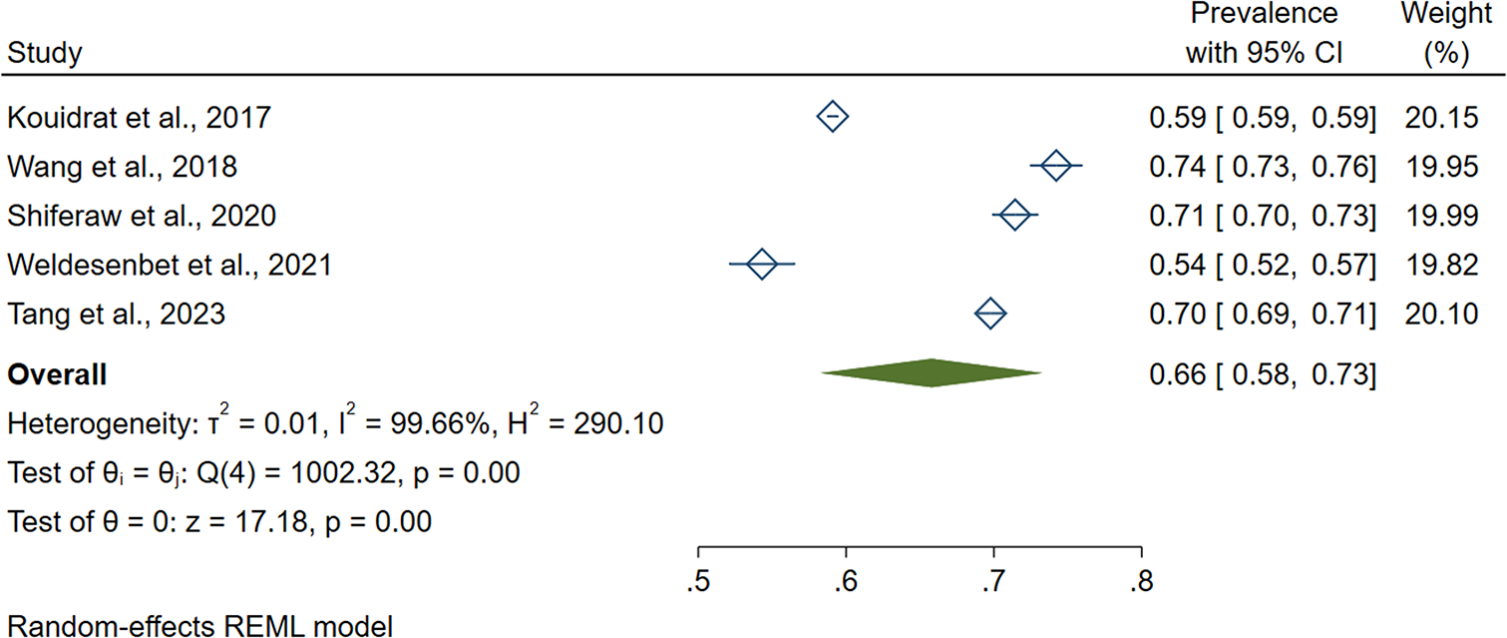
The global pooled prevalence of erectile dysfunction in diabetic patients, 2023

This meta-analysis identified considerable heterogeneity across the studies (I^2^ = 99.66%, p-value = 0.000). As a result, we used a random effect model to estimate the pooled prevalence of erectile dysfunction in diabetic patients. The results of five systematic reviews and meta-analyses studies revealed that the pooled global prevalence of erectile dysfunction in diabetic patients was 0.658 (95CI: 0.583–0.733). (Fig. 2).

### Prediction interval

**Fig. 3:**
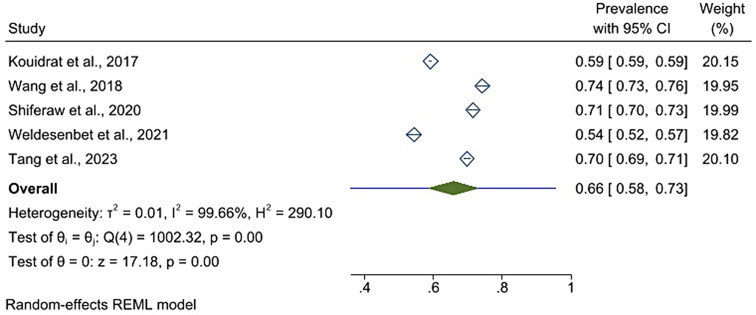
A forest plot shows a prediction interval for pooled prevalence of erectile dysfunction in diabetic patients, 2023

A prediction interval tells us how much variation we can expect in the results of a new study if that study were randomly chosen from the same group of studies used in the current analysis. In other words, it reflects the range of potential outcomes if we include a new study with the same topic. This interval helps us understand how much the combined result might vary depending on the specific new study included [31]. In this umbrella review, the prediction interval for the pooled prevalence of erectile dysfunction is (0.360, 0.955). Thus, if we add a new study, the effect size will fall in the above ranges. (Fig. 3).

### Publication bias

Substantial publication bias was assessed objectively using both Begg’s and Egger’s tests. Both Begg’s and Egger’s tests revealed no publication bias at p-values of 0.9518 and 0.8521, respectively. Besides, a symmetrical distribution of funnel plots was found.

### Subgroup analysis

**Fig. 4:**
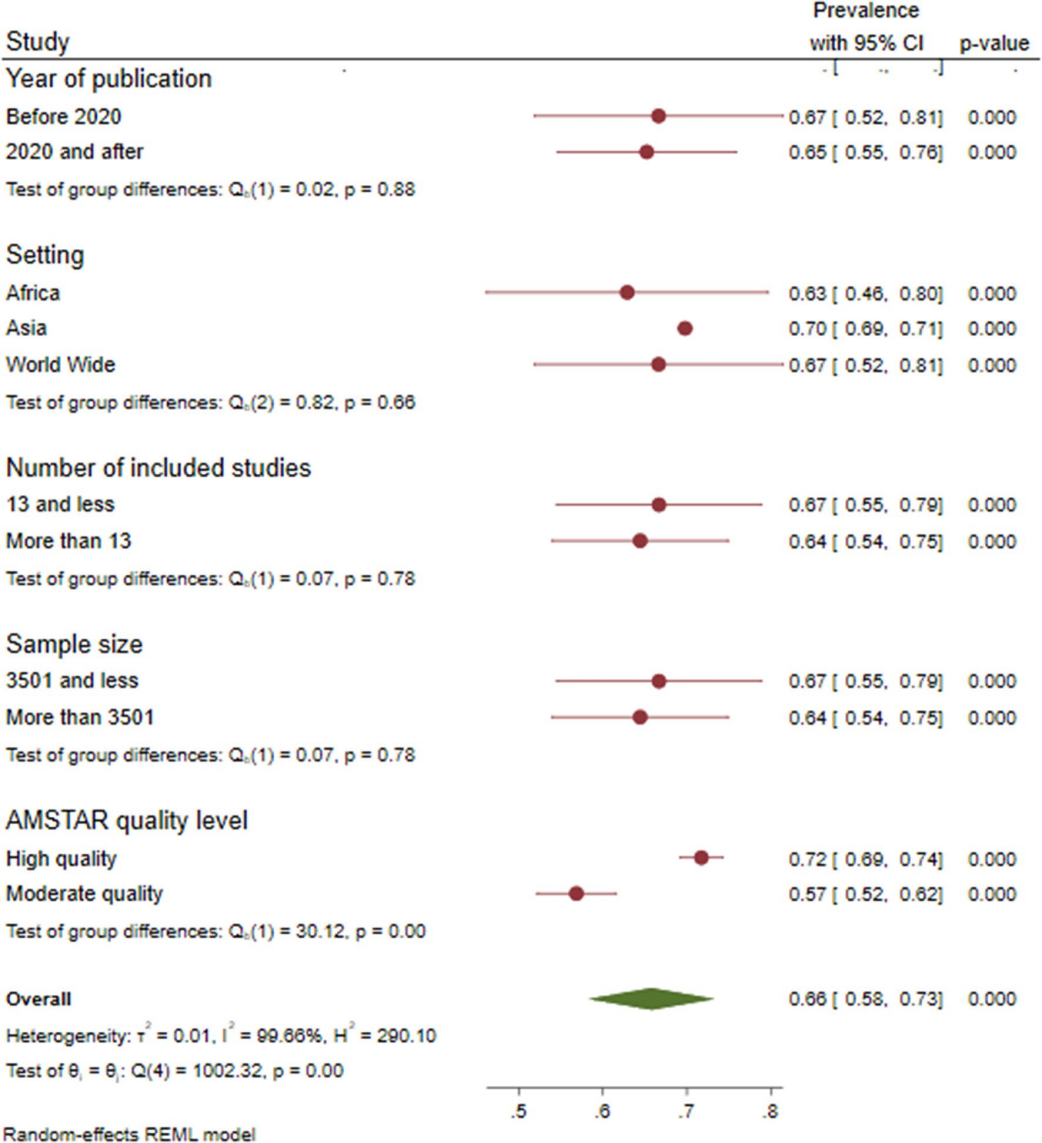
Subgroup analysis of the global pooled prevalence of erectile dysfunction in diabetic patients, 2023

Subgroup analysis was done using publication year, sample size, study quality (AMSTAR 2), setting, and number of included studies. Thus, compared to worldwide prevalence, less prevalence of erectile dysfunction was found in Africa (0.629, 95CI: 0.461–0.797). There was a slight difference in the prevalence of erectile dysfunction among studies done before and after 2020 (0.67%vs. 0.65%). Besides, a high prevalence of ED was found among AMSTAR high-quality studies (0.72). (Fig. 4).

### Sensitivity analysis

Leave-one-out analysis was conducted to explore the influence of a single study on the overall effect size estimate. Leave-one-out meta-analysis omits the corresponding study and performs a meta-analysis on the remaining (n-1 studies). If the cross-ponding study confidence interval doesn’t include the overall effect size estimate (theta), it is declared that the study significantly influences the overall effect size estimate [32]. In this study, the general effect size estimate (theta) is 65.8 and is included within the confidence interval of all studies. Thus, omitting one study does not significantly influence the overall effect size estimate.(Table 2).

**Table 2:** Leave one out meta-analysis to explore the influence one study on the overall pooled prevalence of erectile dysfunction estimation

Omitted study	Prevalence	[95% conf. interval]	*P*-value
Kouidrat et al., [25]	0.675	0.588–0.762	< 0.001
Wang et al., [27]	0.637	0.556–0.718	< 0.001
Shiferaw et al., [28]	0.644	0.554–0.734	< 0.001
Weldesenbet et al., [29]	0.686	0.621–0.751	< 0.001
Tang et al., [26]	0.648	0.554–0.741	< 0.001
Theta	0.658	0.583–0.733	< 0.001

### Risk factors of erectile dysfunction in diabetes patients

Out of the included systematic reviews and meta-analyses, five studies report associated risk factors of erectile dysfunction among diabetic patients. Age (> 40 years) (AOR = 1.95, 95CI: 1.03–3.24), DM duration (> 10years) (AOR = 1.90,95CI: 1.16–2.65), peripheral vascular disease (AOR = 2.74, 95CI: 1.42–4.06) and BMI (> 30 kg/m2) (AOR = 1.07,95CI: 1.01–1.20) were identified as associated risk factors of erectile dysfunction. (Table 3).

**Table 3:** Risk factors of erectile dysfunction in diabetic patients in global, 2023

Risk factors	OR (95CI)	Year	Pooled AOR (95CI)	I^2^ (*P*-value
**Age (> 40 years)**				
Wang et al., [27]	1.77(1.45–2.15)	2018	1.95(1.03–3.24)	89.04(< 0.001)
Shiferaw, Akalu et al., [12]	1.24(1.03–1.51)	2020		
Weldesenbet et al., [29]	4.42(2.83-6.00)	2021		
Tang et al., [26]	1.06(1.03–1.10)	2023		
**DM duration (> 10 years)**				
Wang et al., [27]	1.77(145 − 2.15)	2018	1.90(1.16–2.65)	87.70(< 0.01)
Shiferaw, Akalu et al., [12]	2.63(1.27–5.43)	2020		
Weldesenbet et al., [29]	3.2(1.74–4.66)	2021		
Tang et al., [26]	1.32(1.18–1.47)	2023		
**Peripheral vascular disease**				
Yamada et al., [30]	2.63(1.14–4.91)	2012	2.74(1.42–4.06)	0.00(0.871)
Shiferaw, Akalu et al., [12]	2.85(1.54–5.27)	2020		
**BMI (> 30 kg/m**^**2**^)				
Kouidrat et al., [25]	1.06(0.94–1.20)	2017	1.07(1.01–1.20)	0.00(0.589)
Shiferaw et al., [28]	1.26(0.73–2.16)	2020		

### Age (> 40 years)

**Fig. 5:**
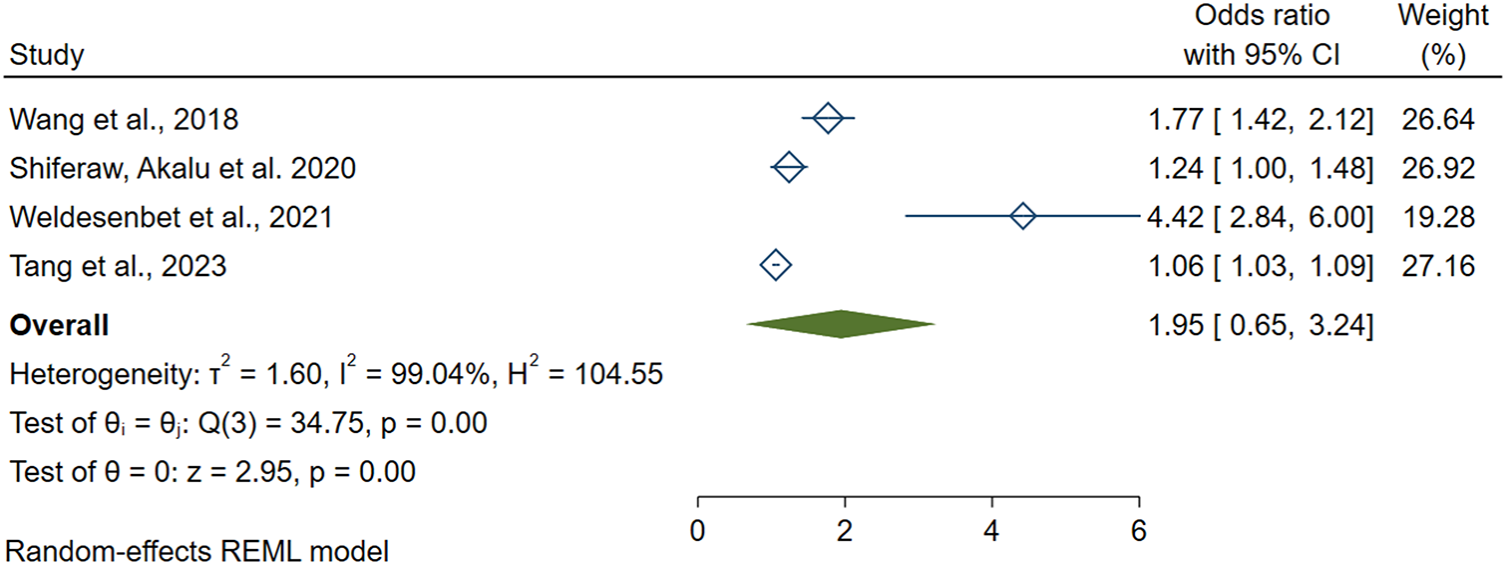
A forest plot shows the pooled estimate of Age (> 40 years) as a risk factor for erectile dysfunction in diabetic patients, 2023

Four systematic reviews and meta-analyses revealed a significant association between age (> 40 years) and erectile dysfunction in diabetic patients. The highest and lowest risk of erectile dysfunction were reported at odds of 4.42 (95CI: 2.83-6.00) and 1.06(95CI: 1.03–1.10), respectively. In this umbrella review, the polled odds of erectile dysfunction among male diabetics aged > 40 years was found to be 1.95 (95CI: 1.03–3.24; I2 = 89.04; *P* < 0.001). (Fig. 5).

#### Publication bias and sensitivity analysis

Regression-based Egger test P-value is < 0.05. There is a publication bias, and trim and fill analysis was done. Thus, 2 studies were added, and the total number of studies became 6. The pooled estimate of AOR of older age becomes (2.13, 95CI: 1.43–3.62). The overall effect size estimate (theta) of the odds ratio for older age was included within the confidence interval of all studies. Thus, omitting one study does not significantly influence the overall effect size estimate.

### DM duration (> 10 years)

**Fig. 6:**
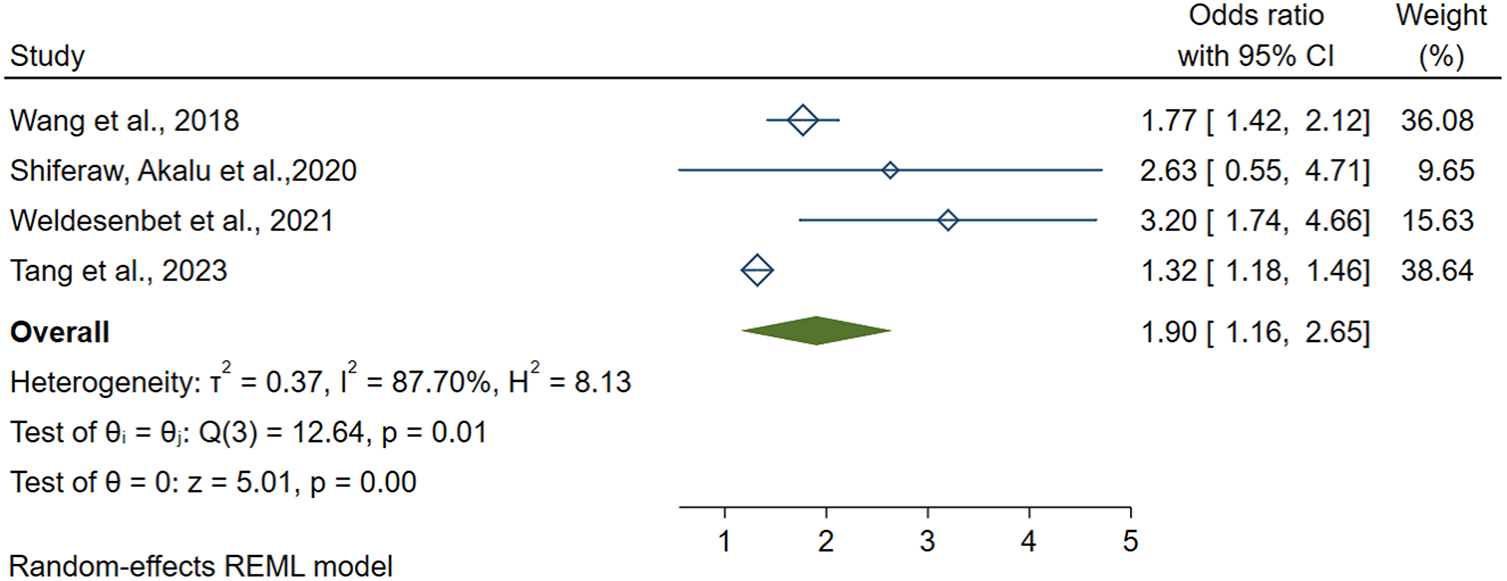
A forest plot shows the pooled estimate DM duration (> 10 years) as a risk factor for erectile dysfunction in diabetic patients, 2023

Four systematic reviews and meta-analyses revealed a significant association between DM duration (> 10 years) and erectile dysfunction in diabetic patients. The highest and lowest risk of erectile dysfunction were reported at odds of 3.2(95CI: 1.74–4.66) and 1.32(95CI: 1.18–1.47), respectively. The polled odds of erectile dysfunction among male patients who live above 10 years with diabetes were 1.90 (95CI: 1.16–2.65; I2 = 87.70; *P* < 0.01). (Fig. 6).

#### Publication bias and sensitivity analysis

Regression-based Egger test P-value is < 0.05. There is a publication bias, and trim and fill analysis was done. Thus, 2 studies were added, and the total number of studies became 6. The pooled estimate of AOR of DM duration becomes (1.52, 95CI: 0.83–2.20). The odds ratio’s overall effect size estimate (theta) for DM duration was 1.90 and included within the confidence interval of all studies. Thus, omitting one study does not significantly influence the overall effect size estimate.

## Discussion

This umbrella review aimed to assess the burden of erectile dysfunction and associated risk factors in diabetic patients in the global context. Thus, the global prevalence of erectile dysfunction in diabetic patients is 65.8% (95CI: 58.3 − 73.3%). Age (> 40 years), DM duration (> 10 years), peripheral vascular disease, and BMI (> 30 kg/m2) were identified as associated risk factors of erectile dysfunction in diabetic patients.

This umbrella review revealed that the pooled prevalence of erectile dysfunction in diabetic patients is 66.8% (58.3 − 73.3%). This finding lower than the study done in India (78.7%) [33], Ethiopia (84.3%) [34] and Japan (90%) [35]. Higher than the study done in Kuwait (31%) [36] and Italy (52.9%) [37]. Alternatively, it aligns with the study done Sri Lanka (62.9%) [38], Hong Kong (63.6%) [39] and Korean (65.4%) [40]. The reason for the variation might be due to variations in study populations, such as the study done in India, Ethiopia, and Japan, which includes a higher proportion of older diabetic patients, who are generally more at risk of developing ED. Conversely, studies conducted in Kuwait and Italy may have included a younger and healthier population, leading to a lower prevalence of ED. Besides, differences in measurement methods might also contribute to the discrepancy. Some studies may use more stringent criteria for diagnosing ED, leading to a higher prevalence, while others may use more lenient criteria, resulting in a lower prevalence. Additionally, standardized questionnaires and diagnostic tools can help ensure consistency in measuring ED prevalence across different studies. The high prevalence of ED in diabetic patients underscores the need for routine screening and early diagnosis of this condition. This is crucial for preventing ED progression and improving patients’ quality of life. Diabetes management should not solely focus on glycemic control but also address ED as a potential complication. This requires a multidisciplinary approach that involves healthcare professionals from various specialties, including diabetologists, urologists, and psychologists.

Consistent with previous findings [41–43] the present study identified increased age as an associated risk factor for erectile dysfunction in diabetic patients. As age increases, the body becomes less efficient at producing and responding to testosterone, the hormone that plays a crucial role in erectile function. This can be further compounded by the vascular damage that occurs in diabetes. High blood sugar levels damage the blood vessels, which can lead to decreased blood flow to the penis, making it difficult to get and maintain an erection [44].

In addition, as the duration of DM after diagnosis increases, the likelihood of getting erectile dysfunction increases. This finding is supported by the previous study [45, 46]. The longer someone has type 2 diabetes, the more likely they are to experience erectile dysfunction (ED), and the more severe the ED tends to be. Studies have shown that around 60% of type 2 diabetes patients who have had the disease for more than two years have ED, and the severity of the ED worsens with increasing duration of the disease [47]. Individuals with diabetes are at higher risk for developing both microvascular and macrovascular complications over time, especially if their blood sugar levels are not well controlled. Poor glycemic control can lead to endothelial dysfunction, which can then block blood flow to the penis and cause erectile dysfunction (ED). Furthermore, as the age of diabetic patient’s increases, the likelihood of developing hypogonadism also increases, which can contribute to erectile dysfunction (ED) [48]. Thus, early screening for ED, along with the initial diagnosis of DM, is crucial for early detection and treatment.

Patients with diabetes mellitus (DM) who also have peripheral vascular disease are more likely to experience erectile dysfunction (ED) than those without peripheral vascular disease. Previous studies have also reported this finding [49–51]. Both diabetes and PVD increase the chances of experiencing erectile dysfunction (ED). When both conditions occur together, the risk of ED becomes even more significant, often leading to more severe and long-lasting problems compared to either condition alone. Diabetes can damage nerves, known as neuropathy, and can interfere with the signals that trigger and maintain an erection [52]. PVD is caused by the buildup of plaque in the arteries. When this plaque formation extends to the arteries supplying blood to the penis, it can significantly reduce blood flow, making it difficult to achieve an erection [53]. The combination of these two conditions can substantially worsen ED. People with diabetes need to manage their blood sugar levels and blood pressure and engage in regular exercise to reduce their risk of ED [54]. Early diagnosis and treatment of ED can significantly improve quality of life.

Body mass index (BMI) greater than 30 kg/m² is a risk factor for erectile dysfunction in diabetic. This finding is consistent with other studies [42, 55]. While the precise connection between obesity and erectile dysfunction (ED) remains unclear, the role of obesity in metabolic syndrome suggests that ED may be caused by underlying pathophysiological processes, including oxidative stress, inflammation, and insulin and leptin resistance. This may contribute to the elevated risk of chronic diseases (hypertension, hyperlipidemia, and diabetes) [56], which can also lead to ED.

This umbrella review builds upon a robust systematic review and meta-analysis encompassing global research on erectile dysfunction (ED). It offers a comprehensive understanding of the burden of ED and its associated risk factors in the global context. The research methodology adheres to the rigorous PRISMA guidelines, guaranteeing the inclusion of high-quality and relevant studies. Furthermore, this review employs the innovative AMSTAR 2 tool to rigorously assess the methodological quality of each included study, providing an additional layer of confidence in the findings. Despite its strengths, the study also has some limitations. First, due to the limited number of studies included, we were unable to examine potential differences in erectile dysfunction between type 1 and type 2 diabetes. Second, although the study aimed for a global context, the included studies did not represent all world regions. This geographical bias limits the generalizability of the pooled results. The high level of heterogeneity among the included studies is also another limitation. Furthermore, the quality of the evidence depends on the quality of the included systematic reviews and meta-analyses.

## Conclusions

The umbrella review found a high global prevalence of erectile dysfunction (ED) in diabetic patients, with two-thirds (65.8%; 95% CI: 58.3–73.3) reporting experiencing ED symptoms. Compared to the worldwide prevalence, a lower prevalence of erectile dysfunction was found in Africa (62.9%; 95% CI: 46.1–79.7). These findings highlight the significant burden of ED faced by diabetic men and underscore the need for global attention to increases awareness for early screening for ED in this population. Age (> 40 years), DM duration (> 10 years), peripheral vascular disease, and BMI (> 30 kg/m2) were identified as associated risk factors of erectile dysfunction in diabetic patients. Addressing various risk factors, including lifestyle modifications (weight management, exercise), glycemic control, and management of underlying conditions like peripheral vascular disease, can play a crucial role in preventing or managing ED. Erectile dysfunction (ED) is a prevalent and often debilitating condition affecting millions of men worldwide. Yet, it remains shrouded in stigma and under-addressed on the global health agenda. Increasing global attention to ED is crucial for improving individual well-being and promoting overall health and development of men’s sexual health.

## Electronic supplementary material

Below is the link to the electronic supplementary material.

Supplementary Material 1

## Data Availability

The datasets used and/or analyzed during the current study available from the corresponding author (TAK) on reasonable request.

## References

[1] Ia A. The likely worldwide increase in erectile dysfunction between 1995 and 2025 and some possible policy consequences. BJU Int. 1999;84:450–6. 10.1046/j.1464-410x.1999.00142.x 10444124

[2] McCabe MP, Sharlip ID, Atalla E, Balon R, Fisher AD, Laumann E, et al. Definitions of sexual dysfunctions in women and men: a Consensus Statement from the Fourth International Consultation on sexual Medicine 2015. J Sex Med. 2016;13(2):135–43. 10.1016/j.jsxm.2015.12.019. 26953828 10.1016/j.jsxm.2015.12.019

[3] Kubin M, Wagner G, Fugl-Meyer AR. Epidemiology of erectile dysfunction. Int J Impot Res. 2003;15(1):63–71. 10.1038/sj.ijir.3900949. 12605242 10.1038/sj.ijir.3900949

[4] Kolluru GK, Bir SC, Kevil CG. Endothelial dysfunction and diabetes: effects on angiogenesis, vascular remodeling, and wound healing. Int J Vasc Med. 2012;2012:918267. 10.1155/2012/918267. 22611498 10.1155/2012/918267 PMC3348526

[5] Msc A. Erectile dysfunction in diabetes: an overview. Int J Innov Stud Med Sci. 2019;3(1):13–4.

[6] Fowler CJ, Ali Z, Kirby RS, Pryor JP. The value of testing for unmyelinated fibre, sensory neuropathy in diabetic impotence. Br J Urol. 1988;61(1):63–7. 10.1111/j.1464-410x.1988.tb09164.x. 3342303 10.1111/j.1464-410x.1988.tb09164.x

[7] Herman WH, Pop-Busui R, Braffett BH, Martin CL, Cleary PA, Albers JW, et al. Use of the Michigan Neuropathy Screening Instrument as a measure of distal symmetrical peripheral neuropathy in type 1 diabetes: results from the Diabetes Control and Complications Trial/Epidemiology of Diabetes Interventions and complications. Diabet Med. 2012;29(7):937–44. 10.1111/j.1464-5491.2012.03644.x. 22417277 10.1111/j.1464-5491.2012.03644.x PMC3641573

[8] Graziottin A. Chapter Female pelvic floor dysfunctions and evidence-based physical therapy| 7. Evidence-Based Physical Therapy for the Pelvic Floor: Bridging Science and Clinical Practice. 2014:243.

[9] Shamloul R, Ghanem H. Erectile dysfunction. Lancet. 2013;381(9861):153–65. 10.1016/s0140-6736(12)60520-0. 23040455 10.1016/S0140-6736(12)60520-0

[10] Phé V, Rouprêt M. Erectile dysfunction and diabetes: a review of the current evidence-based medicine and a synthesis of the main available therapies. Diabetes Metab. 2012;38(1):1–13. 10.1016/j.diabet.2011.09.003. 22056307 10.1016/j.diabet.2011.09.003

[11] Idung AU, Abasiubong F, Ukott IA, Udoh SB, Unadike BC. Prevalence and risk factors of erectile dysfunction in Niger delta region, Nigeria. Afr Health Sci. 2012;12(2):160–5. 10.4314/ahs.v12i2.13. 23056022 10.4314/ahs.v12i2.13 PMC3462533

[12] Shiferaw WS, Akalu TY, Aynalem YA. Prevalence of Erectile Dysfunction in patients with diabetes Mellitus and its association with body Mass Index and Glycated Hemoglobin in Africa: a systematic review and Meta-analysis. Int J Endocrinol. 2020;2020:5148370. 10.1155/2020/5148370. 32411224 10.1155/2020/5148370 PMC7201640

[13] Sharifi F, Asghari M, Jaberi Y, Salehi O, Mirzamohammadi F. Independent predictors of Erectile Dysfunction in type 2 diabetes Mellitus: is it true what they say about risk factors? ISRN Endocrinol. 2012;2012:502353. 10.5402/2012/502353. 22970383 10.5402/2012/502353 PMC3434397

[14] Díaz-Díaz E, León MC, Arzuaga NO, Timossi CM, Díaz RAG, Salinas CA et al. Erectile dysfunction: a chronic complication of the diabetes mellitus. Erectile Dysfunction—Disease-Associated mechanisms and Novel insights into Therapy2012. 2012:69–96.

[15] De Berardis G, Pellegrini F, Franciosi M, Belfiglio M, Di Nardo B, Greenfield S, et al. Longitudinal assessment of quality of life in patients with type 2 diabetes and self-reported erectile dysfunction. Diabetes Care. 2005;28(11):2637–43. 16249532 10.2337/diacare.28.11.2637

[16] Seyam R, Albakry A, Ghobish A, Arif H, Dandash K, Rashwan H. Prevalence of erectile dysfunction and its correlates in Egypt: a community-based study. Int J Impot Res. 2003;15(4):237–45. 12934050 10.1038/sj.ijir.3901000

[17] Elterman DS, Bhattacharyya SK, Mafilios M, Woodward E, Nitschelm K, Burnett AL. The Quality of Life and Economic Burden of Erectile Dysfunction. Res Rep Urol. 2021;13:79–86. 10.2147/rru.S283097. 33634039 10.2147/RRU.S283097 PMC7901407

[18] Aromataris E, Fernandez R, Godfrey CM, Holly C, Khalil H, Tungpunkom P. Summarizing systematic reviews: methodological development, conduct and reporting of an umbrella review approach. JBI Evid Implement. 2015;13(3):132–40. 10.1097/XEB.0000000000000055 26360830

[19] Shea BJ, Reeves BC, Wells G, Thuku M, Hamel C, Moran J, et al. AMSTAR 2: a critical appraisal tool for systematic reviews that include randomised or non-randomised studies of healthcare interventions, or both. BMJ. 2017;358:j4008. 10.1136/bmj.j4008. 28935701 10.1136/bmj.j4008 PMC5833365

[20] Borenstein M, Hedges LV, Higgins JP, Rothstein HR. A basic introduction to fixed-effect and random‐effects models for meta‐analysis. Res Synthesis Methods. 2010;1(2):97–111. 10.1002/jrsm.12 26061376

[21] Higgins JP, Thompson SG, Deeks JJ, Altman DG. Measuring inconsistency in meta-analyses. BMJ. 2003;327(7414):557–60. 12958120 10.1136/bmj.327.7414.557 PMC192859

[22] Ioannidis JP. Interpretation of tests of heterogeneity and bias in meta-analysis. J Eval Clin Pract. 2008;14(5):951–7. 19018930 10.1111/j.1365-2753.2008.00986.x

[23] Higgins JP, Thompson SG. Quantifying heterogeneity in a meta-analysis. Stat Med. 2002;21(11):1539–58. 12111919 10.1002/sim.1186

[24] Egger M, Smith GD, Schneider M, Minder C. Bias in meta-analysis detected by a simple, graphical test. BMJ. 1997;315(7109):629–34. 9310563 10.1136/bmj.315.7109.629 PMC2127453

[25] Kouidrat Y, Pizzol D, Cosco T, Thompson T, Carnaghi M, Bertoldo A, et al. High prevalence of erectile dysfunction in diabetes: a systematic review and meta-analysis of 145 studies. Diabet Med. 2017;34(9):1185–92. 10.1111/dme.13403. 28722225 10.1111/dme.13403

[26] Tang G, Zhang X, Zhu Z. Prevalence of erectile dysfunction and its associated risk factors among Chinese males with diabetes mellitus: a meta-analysis. 2023.

[27] Wang X, Yang X, Cai Y, Wang S, Weng W. High prevalence of Erectile Dysfunction in Diabetic Men with depressive symptoms: a Meta-analysis. J Sex Med. 2018;15(7):935–41. 10.1016/j.jsxm.2018.05.007. 29960629 10.1016/j.jsxm.2018.05.007

[28] Shiferaw WS, Akalu TY, Petrucka PM, Areri HA, Aynalem YA. Risk factors of erectile dysfunction among diabetes patients in Africa: a systematic review and meta-analysis. J Clin Transl Endocrinol. 2020;21:100232. 10.1016/j.jcte.2020.100232. 32685380 10.1016/j.jcte.2020.100232 PMC7358381

[29] Weldesenbet AB, Kebede SA, Tusa BS. Prevalence of erectile dysfunction and its associated factors among patients with diabetes in Ethiopia: a systematic review and meta-analysis. J Int Med Res. 2021;49(2):0300060521993318. 33583238 10.1177/0300060521993318 PMC7890740

[30] Yamada T, Hara K, Umematsu H, Suzuki R, Kadowaki T. Erectile dysfunction and cardiovascular events in diabetic men: a meta-analysis of observational studies. PLoS ONE. 2012;7(9):e43673. 10.1371/journal.pone.0043673. 22962586 10.1371/journal.pone.0043673 PMC3433443

[31] Spineli LM, Pandis N. Prediction interval in random-effects meta-analysis. Am J Orthod Dentofac Orthop. 2020;157(4):586–8. 10.1016/j.ajodo.2019.12.011. 10.1016/j.ajodo.2019.12.011 32241366

[32] Colditz GA, Brewer TF, Berkey CS, Wilson ME, Burdick E, Fineberg HV, et al. Efficacy of BCG vaccine in the prevention of tuberculosis. Meta-analysis of the published literature. JAMA. 2004;271(9):698–702. 8309034

[33] Sondhi M, Kakar A, Gogia A, Gupta M. Prevalence of erectile dysfunction in diabetic patients. Curr Med Res Pract. 2018;8(3):88–91.

[34] Mesfin T, Tekalegn Y, Adem A, Seyoum K, Geta G, Sahiledengle B, et al. Magnitude of erectile dysfunction and associated factors among adult diabetic men on follow-up at Goba and Robe hospitals, Bale Zone, South East Ethiopia: hospital-based cross-sectional study. BMC Endocr Disord. 2023;23(1):236. 10.1186/s12902-023-01489-x. 37880632 10.1186/s12902-023-01489-x PMC10601257

[35] Sasaki H, Yamasaki H, Ogawa K, Nanjo K, Kawamori R, Iwamoto Y, et al. Prevalence and risk factors for erectile dysfunction in Japanese diabetics. Diabetes Res Clin Pract. 2005;70(1):81–9. 10.1016/j.diabres.2005.02.018. 16126126 10.1016/j.diabres.2005.02.018

[36] Al-Hunayan A, Al-Mutar M, Kehinde EO, Thalib L, Al-Ghorory M. The prevalence and predictors of erectile dysfunction in men with newly diagnosed with type 2 diabetes mellitus. BJU Int. 2007;99(1):130–4. 10.1111/j.1464-410X.2006.06550.x. 17026597 10.1111/j.1464-410X.2006.06550.x

[37] Derosa G, Romano D, Tinelli C, D’Angelo A, Maffioli P. Prevalence and associations of erectile dysfunction in a sample of Italian males with type 2 diabetes. Diabetes Res Clin Pract. 2015;108(2):329–35. 10.1016/j.diabres.2015.01.037. 25747572 10.1016/j.diabres.2015.01.037

[38] Nisahan B, Kumanan T, Rajeshkannan N, Peranantharajah T, Aravinthan M. Erectile dysfunction and associated factors among men with diabetes mellitus from a tertiary diabetic center in Northern Sri Lanka. BMC Res Notes. 2019;12(1):210. 10.1186/s13104-019-4244-x. 30953562 10.1186/s13104-019-4244-x PMC6451292

[39] Siu SC, Lo SK, Wong KW, Ip KM, Wong YS. Prevalence of and risk factors for erectile dysfunction in Hong Kong diabetic patients. Diabet Med. 2001;18(9):732–8. 10.1046/j.0742-3071.2001.00557.x. 11606171 10.1046/j.0742-3071.2001.00557.x

[40] Cho NH, Ahn CW, Park JY, Ahn TY, Lee HW, Park TS, et al. Prevalence of erectile dysfunction in Korean men with type 2 diabetes mellitus. Diabet Med. 2006;23(2):198–203. 10.1111/j.1464-5491.2005.01789.x. 16433719 10.1111/j.1464-5491.2005.01789.x

[41] Bacon CG, Hu FB, Giovannucci E, Glasser DB, Mittleman MA, Rimm EB. Association of type and duration of diabetes with erectile dysfunction in a large cohort of men. Diabetes Care. 2002;25(8):1458–63. 10.2337/diacare.25.8.1458. 12145250 10.2337/diacare.25.8.1458

[42] Giugliano F, Maiorino M, Bellastella G, Gicchino M, Giugliano D, Esposito K. Determinants of erectile dysfunction in type 2 diabetes. Int J Impot Res. 2010;22(3):204–9. 20147958 10.1038/ijir.2010.1

[43] Grover SA, Lowensteyn I, Kaouache M, Marchand S, Coupal L, DeCarolis E, et al. The prevalence of erectile dysfunction in the primary care setting: importance of risk factors for diabetes and vascular disease. Arch Intern Med. 2006;166(2):213–9. 16432091 10.1001/archinte.166.2.213

[44] Schardein JN, Hotaling JM. The impact of testosterone on erectile function. Androgens: Clin Res Ther. 2022;3(1):113–24.

[45] Kamenov Z. A comprehensive review of erectile dysfunction in men with diabetes. Exp Clin Endocrinol Diabetes. 2014:141–58. 10.1055/s-0034-1394383 25502583

[46] Widyaningsih N, Ahsani DN, editors. Correlation of Age, Duration of Diabetes Mellitus, HbA1c Levels, and Erectile Dysfunctions in Type II Diabetes Mellitus. 4th International Conference on Sustainable Innovation 2020–Health Science and Nursing (ICoSIHSN 2020); 2021: Atlantis Press.

[47] Yang G, Pan C, Lu J. Prevalence of erectile dysfunction among Chinese men with type 2 diabetes mellitus. Int J Impot Res. 2010;22(5):310–7. 20811390 10.1038/ijir.2010.21

[48] Sihite AP, Pramesemara IGN, Surudarma IW. Relationship of type 2 diabetes Mellitus Duration with the occurrence of Erectile Dysfunction at Puskesmas Denpasar Barat I.

[49] Meena BL, Kochar DK, Agarwal TD, Choudhary R, Kochar A. Association between erectile dysfunction and cardiovascular risk in individuals with type-2 diabetes without overt cardiovascular disease. Int J Diabetes Dev Ctries. 2009;29(4):150–4. 10.4103/0973-3930.57345. 20336196 10.4103/0973-3930.57345 PMC2839128

[50] Gandaglia G, Briganti A, Jackson G, Kloner RA, Montorsi F, Montorsi P, et al. A systematic review of the association between erectile dysfunction and cardiovascular disease. Eur Urol. 2014;65(5):968–78. 10.1016/j.eururo.2013.08.023. 24011423 10.1016/j.eururo.2013.08.023

[51] Yuan P, Ma D, Zhang Y, Gao X, Wang J, Li R, et al. Analysis of cardiovascular risks for erectile dysfunction in Chinese patients with type 2 diabetes mellitus lacking clinical symptoms of cardiovascular diseases. Transl Androl Urol. 2020;9(6):2500–9. 10.21037/tau-20-999. 33457224 10.21037/tau-20-999 PMC7807359

[52] Kizilay F, Gali HE, Serefoglu EC. Diabetes and sexuality. Sex Med Reviews. 2017;5(1):45–51. 10.1016/j.sxmr.2016.07.002 27544297

[53] Gerber RE, Vita JA, Ganz P, Wager CG, Araujo AB, Rosen RC, et al. Association of peripheral microvascular dysfunction and erectile dysfunction. J Urol. 2015;193(2):612–7. 25196657 10.1016/j.juro.2014.08.108

[54] Duca Y, Calogero AE, Cannarella R, Giacone F, Mongioi LM, Condorelli RA, et al. Erectile dysfunction, physical activity and physical exercise: recommendations for clinical practice. Andrologia. 2019;51(5):e13264. 30873650 10.1111/and.13264

[55] Pizzol D, Smith L, Fontana L, Caruso MG, Bertoldo A, Demurtas J, et al. Associations between body mass index, waist circumference and erectile dysfunction: a systematic review and META-analysis. Reviews Endocr Metabolic Disorders. 2020;21:657–66. 10.1007/s11154-020-09541-0 32002782

[56] Liu Y, Hu X, Xiong M, Li J, Jiang X, Wan Y, et al. Association of BMI with erectile dysfunction: a cross-sectional study of men from an andrology clinic. Front Endocrinol (Lausanne). 2023;14:1135024. 10.3389/fendo.2023.1135024. 37065736 10.3389/fendo.2023.1135024 PMC10101565

