# Supplementary material for: The global burden of erectile dysfunction and its associated risk factors in diabetic patients: an umbrella reviews
Source: BMC Public Health. 2024 Oct 14;24:2816. doi: 10.1186/s12889-024-20300-7 (PMC11472474; doi:10.1186/s12889-024-20300-7)
Supplement: Supplementary file 1 — Supplementary Material 1 [file 12889_2024_20300_MOESM1_ESM.docx]

| No. | AMSTAR2 criteria | Studies | | | | | | |
| --- | --- | --- | --- | --- | --- | --- | --- | --- |
|  |  | Kouidrat et al., 2017 | Shiferaw, Akalu et al. 2020 | Tang et al., 2023 | Wang et al., 2018 | Weldesenbet et al., 2021 | Shiferaw et al., 2020 | Yamada et al., 2012 |
| 1 | Did the research questions and inclusion criteria for the review include the components of PICO? | Yes | Yes | Yes | Yes | No | Yes | No |
| 2 | Did the report of the review contain an explicit statement that the review methods were established prior to the conduct of the review and did the report justify any significant deviations from the protocol? | Yes | Yes | Yes | Yes | yes | Yes | Yes |
| 3 | Did the review authors explain their selection of the study designs for inclusion in the review? | Yes | Yes | Yes | Yes | yes | Yes | Yes |
| 4 | Did the review authors use a comprehensive literature search strategy? | Yes | Yes | Yes | Yes | yes | Yes | Yes |
| 5 | Did the review authors perform study selection in duplicate? | Yes | Yes | Yes | Yes | yes | Yes | Yes |
| 6 | Did the review authors perform data extraction in duplicate? | No | Yes | Yes | Yes | yes | Yes | Yes |
| 7 | Did the review authors provide a list of excluded studies and justify the exclusions? | Yes | Yes | Yes | Yes | yes | Yes | Yes |
| 8 | Did the review authors describe the included studies in adequate detail? | No | Yes | Yes | Yes | yes | Yes | Yes |
| 9 | Did the review authors use a satisfactory technique for assessing the risk of bias (RoB) in individual studies that were included in the review? | Yes | Yes | Yes | Yes | yes | Yes | Yes |
| 10 | Did the review authors report on the sources of funding for the studies included in the review? | Yes | Yes | Yes | Yes | yes | Yes | Yes |
| 11 | If meta-analysis was performed did the review authors use appropriate methods for statistical  combination of results? | Yes | Yes | Yes | Yes | yes | Yes | Yes |
| 12 | If meta-analysis was performed, did the review authors assess the potential impact of RoB in  individual studies on the results of the meta-analysis or other evidence synthesis? | Yes | No | Yes | Yes | No | Yes | No |
| 13 | Did the review authors account for RoB in individual studies when interpreting/ discussing the results of the review? | Yes | Yes | Yes | Yes | yes | Yes | No |
| 14 | Did the review authors provide a satisfactory explanation for, and discussion of, any  heterogeneity observed in the results of the review? | Yes | Yes | Yes | Yes | yes | Yes | Yes |
| 15 | If they performed quantitative synthesis did the review authors carry out an adequate  investigation of publication bias (small study bias) and discuss its likely impact on the results of the review? | Yes | Yes | Yes | Yes | yes | Yes | Yes |
| 16 | Did the review authors report any potential sources of conflict of interest, including any funding hey received for conducting the review? | Yes | Yes | Yes | Yes | yes | Yes | Yes |
|  | **Quality of review** | **Moderate** | **High** | **High** | **High** | **Moderate** | **High** | **Low** |
